# Inducing Collagen Synthesis by a Combination of an Integrin Receptor-Stimulating Collagen-Based Peptide and Chlorella Vulgaris Extract

**DOI:** 10.34172/apb.46552

**Published:** 2026-06-27

**Authors:** Marjan Gholami, Seyed Shahriar Arab, Mehrdad Behmanesh

**Affiliations:** ^1^Department of Genetics, Faculty of Biological Sciences, Tarbiat Modares University, Tehran, Iran; ^2^Department of Biophysics, Faculty of Biological Sciences, Tarbiat Modares University, Tehran, Iran

**Keywords:** *Chlorella vulgaris*, Collagen-based peptide, Collagen synthesis, Fibroblast migration, Integrin, Wound healing

## Abstract

**Purpose:**

Collagen synthesis is crucial for effective wound healing, yet natural repair is often a slow process. We developed a 10-amino-acid integrin-binding peptide (YFPGERGRPG) and combined it with *Chlorella vulgaris* (*C. vulgaris*) extract to investigate their combined effects on enhancing fibroblast-mediated collagen production and tissue repair.

**Methods:**

*C. vulgaris* was prepared by sonication, centrifugation, and filtration. Human dermal fibroblasts (HDF) were treated with the synthetic peptide and algal extract, and their effects were assessed using 3-(4,5-dimethylthiazol-2-yl)-2,5-diphenyltetrazolium bromide (MTT) assay, scratch wound assay, and gene expression analysis. *In vivo* wound healing was evaluated in a rat excisional wound model through wound closure, Masson’s Trichrome staining, and Western blotting.

**Results:**

The results indicated that the combination of synthetic peptide and *C. vulgaris* extract had significant effects on collagen synthesis compared to individual treatments (*P*<0.05). The scratch assay demonstrated quicker wound closure, and gene expression analysis confirmed the upregulation of collagen type I alpha 1 (*COL1A1*) (*P*<0.05). *In vivo*, topical application in a full-thickness rat skin wound model had significant effects on collagen deposition, wound contraction, and epithelial regeneration, compared to individual treatments and standard collagen dressings. Histological analyses confirmed increased collagen density, consistent with gene expression and functional outcomes.

**Conclusion:**

Notably, the combined effects of the peptide and extract were observed at both molecular and tissue levels, highlighting the potential of this combinatorial approach to influence wound repair. These results support the therapeutic potential of integrating bioactive peptides with microalgal extracts to investigate their effects on extracellular matrix (ECM) remodeling for regenerative therapies. Further studies are required to elucidate underlying mechanisms and assess clinical translation.

## Introduction

 Chronic and non-healing skin wounds are a growing global health concern, particularly among elderly individuals, patients with diabetes, and those with vascular or immune-related disorders. These wounds significantly impair quality of life and impose a substantial economic burden on healthcare systems. In the United States alone, the estimated annual cost of chronic wound care exceeds $25 billion, highlighting the pressing need for effective therapeutic interventions.^1^

 Effective wound healing requires dynamic interactions between cells and the extracellular matrix (ECM), particularly collagen, which provides both structural support and signaling cues. In the dermis, fibroblasts are responsible for producing types I and III collagen, which account for 80–90% of total collagen content.^2^ Beyond their structural roles, ECM components carry adhesive motifs that interact with various cell surface receptors.^3,4^

 Integrins represent one of the major families of collagen-binding receptors.^3^ These transmembrane glycoproteins mediate cell adhesion, migration, proliferation, and survival in response to ECM signals.^5^ By sensing environmental changes, integrins regulate key cellular responses during inflammation and tissue repair.^6^ Specifically, collagen-binding integrins such as α1β1, α2β1, α10β1, and α11β1 recognize peptide motifs within the collagen triple helix.^4^ Based on cell culture experiments, it has been suggested that α2β1 integrins play a role in various aspects of matrix remodeling, including the formation of collagen fibrils,^7^ even *in vivo*.^8^

 A specific collagen motif, hexapeptide the Gly-Phe-Hyp-Gly-Glu-Arg (GFOGER) motif, recognized by the integrin α2β1 I domain, was found in the α1 chain of type I collagen.^9^ However, the sequence GAOGER in collagen III represents another α2β1 recognition sequence.^5^ Owing to their central roles in physiology and disease pathobiology, integrins have become major therapeutic targets in biotechnology and pharmaceutical research.^10^

 From the past to the present, several natural materials have been experimentally utilized in traditional medicine to treat skin disorders and wound injuries, aiming to enhance healing and reduce the cost of raw materials. Recent studies have demonstrated that marine resources, including marine algae, are rich sources of biologically active compounds with a range of beneficial properties.^11^ *Chlorella vulgaris (C. vulgaris)* is* a* green unicellular microalga discovered in 1890 by Martinus Willem Beijerinck. It grows in fresh water and is often consumed as a health supplement in some countries, notably Japan.^12^ *C. vulgari*s rich in natural products such as proteins, fatty acids, nucleic acids, and secondary metabolites.^11^ Chlorella growth factor (CGF), a unique group of substances found in the nucleus of Chlorella, exhibits numerous biological functions, including antitumor, anti-inflammatory, and antioxidant activities.^12^ Although several studies are in progress to examine the biological effects of *C. vulgaris*, studies using it for wound healing are still scarce in the literature.

 Recently, by understanding how certain intracellular anabolic pathways contribute to protein expression,^12^ researchers have explored designing molecules that disrupt or enhance these interactions via cell surface receptors, which have long been a potential therapeutic target.^4^ Consequently, the synthesis of collagen-mimetic materials has primarily focused on peptides, and strategies to generate cell-responsive synthetic materials have included the chimeric integration of bioactive ECM sites into synthetic peptides or polymers.^2^ Synthetic peptides are integral to protein science, and the collagen field is no exception. Peptides containing the sequence GFOGER were initially selected as potential binding sites due to their high affinity for the isolated I-domain and ability to support α2β1-mediated cell adhesion.^3^

 Modulating these interactions has emerged as a promising strategy to improve wound healing. In this context, our study focused on a synthetic integrin-binding peptide in combination with *C. vulgaris *extract to investigate their effects on collagen remodeling and tissue repair. Although both bioactive peptides and microalgal extracts have independently demonstrated regenerative potential, their combined application has not been systematically explored. The integrin-targeting peptide used in this study was designed to enhance cell–matrix interactions and stimulate collagen-related signaling pathways. In parallel, *C. vulgaris *extract contains bioactive compounds, including proteins, polysaccharides, and antioxidants, which have been reported to support ECM production and cellular viability. We therefore hypothesized that the simultaneous modulation of integrin-mediated adhesion signaling and provision of pro-regenerative bioactive molecules could exert a complementary effect on wound repair.

## Materials and Methods

###  Peptide Design 

####  Data Collection 

 To identify collagen motifs involved in interaction with the integrin α2 receptor, the three-dimensional structure of the collagen–integrin α2 complex was obtained from the RCSB Protein Data Bank (PDB ID: 1DZI), and interaction analysis was performed using UCSF Chimera and LigPlot+ software. Previous studies have indicated that glutamic acid (Glu) plays a critical role in collagen–integrin binding.^9^ Therefore, residues 8–14 were selected as the interacting segment.

 This structure was considered a reference for control peptide-receptor interactions. CFinder (Contact Finder) online tools were also used to identify interface residues in a protein complex (http://bioinf.modares.ac.ir/software/nccfinder/CFinder).

####  Peptide Modeling 

 YASARA software has been used to model the desired peptide sequences. Besides, Osprey software was utilized to introduce mutations with minimized energy levels in a protein. This analysis systematically evaluates various changes to determine the optimal configuration for minimizing energy. Results from Osprey suggested replacing Pro at position 14 with either Arg or Lys. For advanced structural insights, Nanome virtual reality software was used to visualize the protein in 3D. This revealed that Gly 7 was adjacent to Tyr 157 of the receptor. We anticipated improved interactions by replacing Gly with an amino acid possessing a phenolic ring (e.g., Phenylalanine or Tyrosine). A combined mutation strategy was expected to strengthen peptide-receptor binding. Finally, we generated four modified peptides (P1, P2, P13, and P23) in contact with integrin α2 and used them for further investigations.

####  Molecular Dynamics Simulation

 Molecular dynamics (MD) simulations of the final five systems—one control and four modified peptides—were carried out using GROMACS 2022.6 with the OPLS force field. Water molecules were added via the SPC single point charge model. The systems were then neutralized using the Genion tool with counter ions. Energy minimization was performed with the Steepest Descent algorithm. Subsequently, the systems were equilibrated to temperature (300 K) and pressure (1 bar) using the V-rescale and Parrinello-Rahman algorithms, respectively. Finally, each system underwent 100 ns production runs, with trajectories recorded every two ps.

 The root mean square deviation (RMSD) and Radius of Gyration (Rg) analysis were carried out to investigate the system’s overall stability using the GROMACS program. The GROMACS H-bond program was also used to determine the number of hydrogen bonds (HB) between the receptor and its peptide ligands. Moreover, the molecular mechanics Poisson–Boltzmann surface area (MM-PBSA) method was used to calculate the binding free energy of receptor peptides through the g-mmpbsa tools.

 The peptide P13 was synthesized using solid-phase peptide synthesis following Fmoc Chemistry by ProteoGenix Inc., France. The complete sequence of the synthesized peptide was YFPGERGRPG.

###  C. Vulgaris Extraction

*C. vulgaris* was purchased from Parsian Microalgae Co. (Tehran, Iran). Dry biomass was extracted in distilled water (8% v/v) at 4°C by sonication, using five pulses of 5 minutes with a 1-minute interval, under a frequency of 20 kHz at a power of 200 W. After extraction, the aqueous *C. vulgaris *extract was centrifuged, and the supernatant was collected. The total protein concentration of the extract was measured using a NanoDrop spectrophotometer (Thermo Scientific, USA) at 280 nm. The extract was diluted to desired protein concentrations (0.625 to 80 µg/mL) for cell treatment assays. All concentrations used in experiments were based on protein content. The dosage applied in cellular assays was selected based on a preliminary cytotoxicity screening to determine a non-toxic concentration range.

###  Cell Culture

 Human dermal fibroblast (HDF) cells were purchased from the Pasteur Institute (Tehran, Iran) and received in Nunc T-75 cell culture flasks. HDF cells were cultured in Dulbecco’s Modified Eagle Medium (DMEM), supplemented with 10% (v/v) fetal bovine serum (FBS) and 100 units of penicillin and 100 micrograms/ml of streptomycin. All cultured cells were incubated at 37°C in a humidified atmosphere of 5% CO₂. Cells were harvested at 80-90% confluency.

###  Cell Viability Assay

 Cells were seeded into 96-well plates at a density of 5 × 10³ cells/well. After overnight incubation to allow cell adhesion, the used media were replaced with fresh media containing different concentrations of either the synthetic peptide (0.3125, 0.625, 1.25, 2.5, 5, and 10 µg/ml) or *C. vulgaris* extract (0.625, 1.25, 2.5, 5, 10, 20, 40, and 80 µg/ml), and cells were incubated further. Based on the results of this first phase, in which the cytotoxicity of each compound was independently evaluated, concentrations that showed no significant cytotoxicity were selected for the second phase of the study. In this phase, a combination of non-toxic concentrations of the peptide (0.3125, 0.625, 1.25, and 2.5 µg/ml) and *C. vulgaris* extract (0.625, 1.25, 2.5, 5, and 10 µg/ml) was tested in co-treatment assays.

 The test was performed at three different time points (24 h, 48 h, and 72 h). At the end of the incubation, the medium was removed, and 20 µL of 3-(4,5-dimethylthiazol-2-yl)-2,5-diphenyltetrazolium bromide (MTT, 5 mg/ml) solution was added to each well, which is reduced by metabolically active cells to insoluble purple formazan crystals. The cells were further incubated for 4 hours at 37°C. The cells were dissolved in 150 µL of dimethyl sulfoxide (DMSO), and the absorbance of formazan at 570 nm was measured using an enzyme-linked immunosorbent assay (ELISA) reader. The optical density reflects the level of cell metabolic activity. Experiments were repeated independently in triplicate. Cell viability was calculated using the formula A_t_/A_c_ × 100, where A_t_ and A_c_ are the absorbance of the treatment and control groups, respectively.

###  Gene Expression Assay

####  RNA Extraction

 Based on the results of the cell viability assay results, non-toxic concentrations of the treatments were selected for subsequent gene expression analysis. Therefore, RNA extraction was only performed on a limited number of experimental conditions.

 Following a 48-hour treatment, total cellular RNA was isolated from HDFs exposed to the following groups: **a)** synthetic peptide (0.625 µg/ml), **b)** synthetic peptide (1.25 µg/ ml), **c)***C. vulgaris* extract (2.5 µg/ ml), **d)** a combination of synthetic peptide (0.625 µg/ml) and *C. vulgaris *extract (2.5 µg/ml), and **e)** a combination of synthetic peptide (1.25 µg/ml) and *C. vulgaris *extract (2.5 µg/ml).

 RNA was extracted using TriZol reagent (Invitrogen) according to the manufacturer’s instructions. The concentration and purity of RNA were measured using a NanoDrop spectrophotometer, and only samples with OD260/OD280 ratios between 1.9 and 2.0 were used for downstream analysis. RNA integrity was confirmed 1% agarose gel electrophoresis, stained with ethidium bromide, and visualized under UV light.

####  cDNA Synthesis and Quantitative Real-Time Polymerase Chain Reaction Analysis

 Quantitative real-time polymerase chain reaction (qPCR) analysis of relative mRNA expression of *COL1A1*, collagen type I alpha2 (*COL1A2)*, collagen type 3 alpha1 (*COL3A1)*, and beta-2 microglobulin (*β2M*) as the housekeeping gene was performed using specific primers. The primers used to amplify different genes are listed in Table 1.

**Table 1:** Primer sequences were used in this study to investigate gene expression changes using the qPCR technique.

**Gene**	**Transcript**	**Primer Name**	**Primer Sequence (5'→3’)**	**Amplicon length(bp)**
*ITGA2*	NM_002203.4	Forward	CCCTGCCCTTCCCTCATAGAT	141
		Reverse	AATTAACCCCACCTGTGTCTTTG	
*COL1A1*	NM_000088.4	Forward	CTGACTGGGAGAGTGGAGAGT	162
		Reverse	TGTCCTTGGGGTTCTTGCTG	
*COL1A2*	NM_000089.4	Forward	AACTTTGCTGCTCAGTATGATG	126
		Reverse	AGCATGTCCTTGGAAACCTTG	
*COL3A1*	NM_000090.4	Forward	AGGACTGACCAAGATGGGAAC	96
		Reverse	AGGGGAGCTGGCTACTTCTC	
*Β 2M*	NM_004048.4	Forward	AGATGAGTATGCCTGCCGTGT	106
		Reverse	TGCGACATCTTCAAACCTCCA	

 cDNA synthesis from 3 µg of total RNA was performed using the Reverse Transcriptase Viragene Kit (Tehran, Iran) according to the manufacturer’s protocol. Then, the cDNA was used as the template for qPCR. Relative qPCR was performed on all samples analyzed in duplicate using SYBR Green in the Real-Time PCR (ABI Step One Plus). The thermocycling protocol consisted of an initial denaturation at 95°C for 15 minutes, followed by 40 cycles of denaturation at 95°C for 15 seconds and combined annealing/extension at 60°C for 60 seconds. The relative changes in gene expression were calculated using threshold cycle (C_T_) values that were first normalized to the *β2M* housekeeping gene and using the ΔCT value of control samples as the calibrator. The obtained data were analyzed using the 2^-ΔΔCT^ method. *P* < 0.05 was considered statistically significant.

###  Scratch Assay

 HDF cells were seeded at a density of 1.5 × 10⁵ cells per well in a 12-well plate and cultured overnight until they formed a confluent monolayer, then subjected to serum starvation for 24 hours. The monolayer was subsequently scratched with a pipette tip to create an approximate 1-mm-wide wound area and washed twice with 1 ml phosphate-buffered saline (PBS) to remove floating cells. After the scratch, 2 mL DMEM was added to each well. To assess the effect of treatment groups on fibroblast migration, cells were incubated with a) synthetic peptide (0.625 µg/ml), b) *C. vulgaris* extract (2.5 µg/ml), and c) a combination of synthetic peptide (0.625 µg/ml) with algae extract (2.5 µg/ml) for varying durations (from 0 hours to 48 hours), in the presence of mitomycin C (5 µg/ml) to inhibit cell proliferation. Micrographs of the wound edges were captured using a microscope at 0, 12, 24, and 48 hours, and analyzed with ImageJ software (National Institutes of Health, Bethesda, MD, USA). The cell migration rate was calculated as Lt/Lc × 100% (where Lt and Lc represent the lengths between the two cell edges in the treatment and control groups, respectively). All experiments were performed independently in triplicate.

###  In Vivo Wound Healing Assay

####  Animal Grouping and Treatment Schedule

 A total of 10 eight-week-old male Wistar rats (170 ± 10 g) were obtained from the Safadasht Animal House Centre (Safadasht-Iran). Animals were housed under standard laboratory conditions (25 ± 2˚C, 50–70% relative humidity, 12 h light/dark cycle) with free access to food and water and acclimatized for one week before experimentation.

 Under anesthesia, four full-thickness excisional wounds were created on the dorsal surface of each rat. A within-animal experimental design was employed, whereby each animal received multiple treatments on separate wounds to minimize inter-animal variability. Five rats were allocated to Group A and five to Group B (n = 10 total animals). In Group A, the four wounds of each rat were treated topically with: (i) 0.4 mg/mL *C. vulgaris* extract, (ii) 0.625 mg/mL synthetic peptide, (iii) a combination of 0.625 mg/mL synthetic peptide and 0.4 mg/mL *C. vulgaris* extract, and (iv) Vaseline as vehicle control. In Group B, the four wounds of each rat received: (i) SORBACT® wound dressing (positive control), (ii) no treatment (negative control), (iii) the combination of 0.625 mg/mL synthetic peptide and 0.4 mg/mL *C. vulgaris* extract, and (iv) Vaseline as vehicle control. Treatment allocation to wound sites was standardized according to anatomical position to avoid positional bias.

 The concentrations used for topical* in vivo* application were guided by the *in vitro* cytocompatibility and efficacy data. Preliminary screening in the wound model was performed to identify concentrations that produced measurable biological effects without observable adverse reactions. Lower concentrations that did not outperform the vehicle control were excluded.

 All experimental procedures were approved by the Research Ethics Committees of Tarbiat Modares University (Approval No.: IR.MODARES.REC.1400.281).

####  Wound Creation and Wound Size Assessment

 Before surgery, rats were anesthetized with a ketamine-xylazine combination (ketamine 100 mg/kg and xylazine 10 mg/kg). Subsequently, the rats’ back hair was shaved and disinfected with 70% ethanol. Four full-thickness round excision wounds (15 mm in diameter) were created on the dorsal region of the animal’s back using a punch plier. At the end of the surgical procedure, the animals were kept in cages placed near a heating apparatus until they fully recovered from anesthesia. The wounds were photographed and measured on days 0, 3, 6, 9, 11, and 14 post-wounding using a digital camera to evaluate the healing potential of each treatment. The wound area and closure rate were analyzed using ImageJ software. The measurements were performed in triplicate, and the mean values of consecutive tracings were computed and expressed as a percentage of closure from the original wound (day 0 = 100%).

###  Histology Evaluation

####  Tissue Sample Collection

 Rats from both groups were sacrificed by intraperitoneal injection of an overdose of ketamine-xylazine on days 3, 6, 9, 11, and 14 post-wounding, and skin tissue samples from the wound site were collected from all the rats for histological and immunoblotting analysis.

####  Histopathological Analysis

 Excised skin samples were fixed in a 10% formalin buffered solution (pH 7.4) for 48 h, dehydrated through a graded series of ethanol, followed by histological processing through paraffin embedding and sectioning. Deparaffinized sections (5 µm thick) were stained with Masson’s Trichrome staining according to the manufacturer’s protocol and assessed under a light microscope equipped with a camera. Three images per field were captured to obtain the mean. The images were stored and subjected to measuring collagen density with the aid of ImageJ software.

###  Western Blot Analysis

 A second set of specimens of wound tissue samples was collected in sterile microtubes, immediately snap-frozen in liquid nitrogen, and stored at −80°C. Approximately 500 mg of frozen tissue was homogenized in lysis buffer, and total protein was extracted following centrifugation. Protein concentration was determined using the Bradford assay (Bio-Rad, USA) with bovine serum albumin (BSA) as the standard.

 Equal amounts of protein (25 µg per lane) were denatured, separated by 12.5% sodium dodecyl sulfate–polyacrylamide gel electrophoresis (SDS-PAGE), and transferred to polyvinylidene difluoride (PVDF) membranes. Membranes were blocked with 5% skim milk in Tris-buffered saline with Tween 20 (TBST), then incubated overnight at 4°C with primary antibodies against *COL1A1* (mouse monoclonal, 1:1000, Santa Cruz Biotechnology, USA) and β-actin (mouse monoclonal, 1:1000, Santa Cruz). After washing, membranes were incubated with appropriate horseradish peroxidase (HRP)-conjugated secondary antibodies (1:4000, Santa Cruz) and developed using a chemiluminescence detection system. All procedures were performed according to the manufacturer’s protocol.

 Finally, the protein bands were analyzed using ImageJ software and radiography film (Fuji Film, Japan). Immunoblot data were normalized to β-actin protein levels. All experiments were performed independently in duplicate.

###  Statistical Analyses

 All data were presented as mean ± standard deviation (SD). Statistical analyses were performed using GraphPad Prism software (GraphPad Software, San Diego, CA, USA). The normality of the data was assessed using the Shapiro-Wilk test. Since the data distribution was found to be normal, the parametric test was deemed an appropriate statistical method for the analysis. Tukey’s post-hoc test was used for comparisons among treatment groups. Each wound within a rat was treated as a nested observation to account for intra-animal correlation. All statistical tests were two-tailed, and a *P* < 0.05 was considered statistically significant.

## Results

###  Computational Simulation Analysis

####  Comparative MD Simulations Revealed Differential Stability Among Peptide–Receptor Complexes.

 RMSD analysis over 100 ns demonstrated that complexes containing the P1 and P2 peptides remained structurally stable, exhibiting minimal deviations (< 0.1 nm), whereas the other three systems showed larger fluctuations (0.15–0.35 nm). Notably, the complex with the P23 peptide displayed the highest instability, with RMSD values exceeding 0.6 nm (Figure 1A).

**Figure 1:**
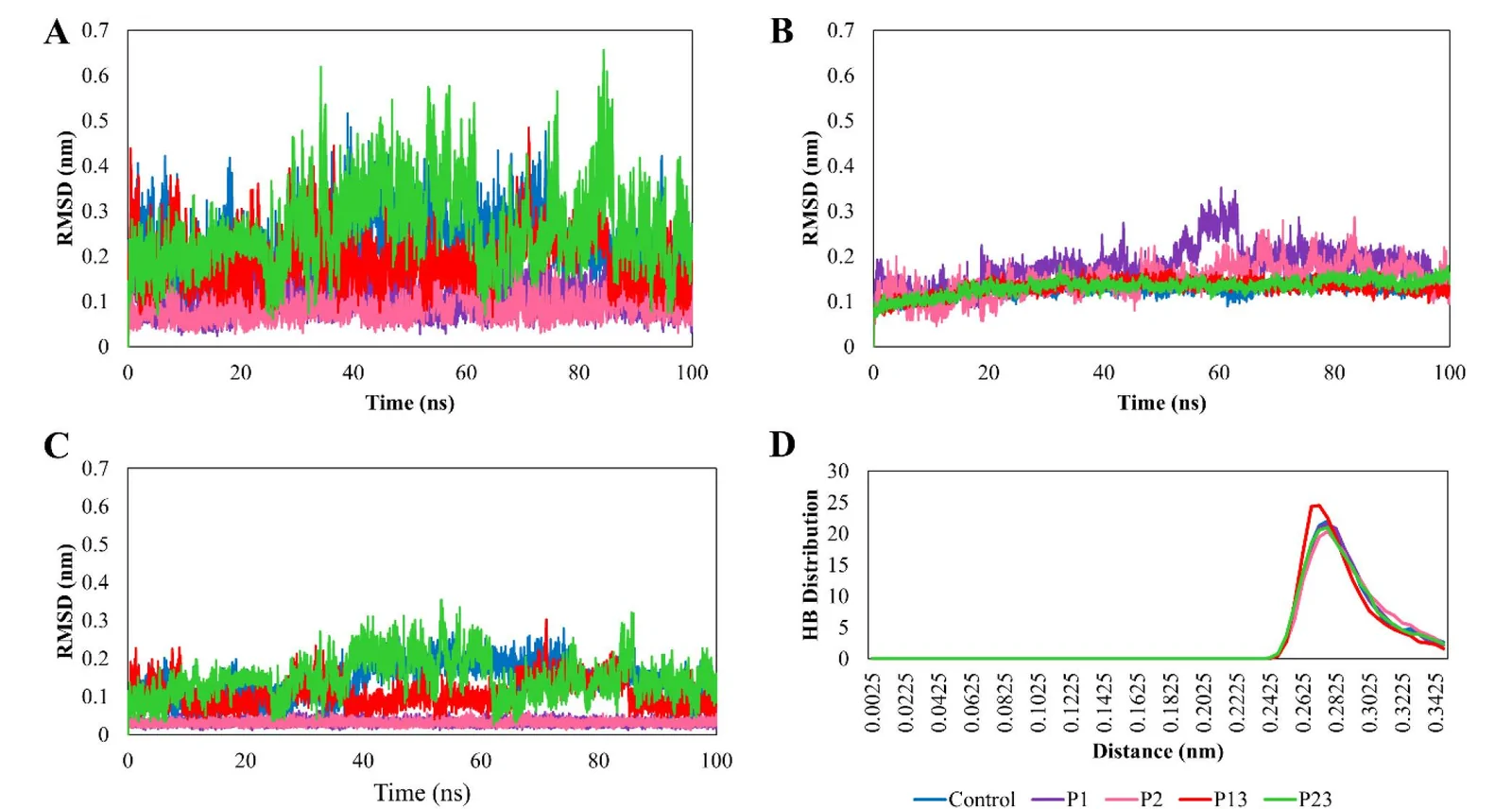
RMSD profiles of (A) protein-peptide complex, (B) the protein alone, and (C) the peptides during 100 ns MD simulation. (D) Distribution of HB distances during the last 30 ns of the simulation. Systems containing control, P1, P2, P13, and P23 peptides are represented in different colors: blue, purple, pink, red, and light green, respectively

####  Protein and Peptide Components Exhibited Stable Conformations After Equilibration.

 Separate RMSD analyses of the proteins and peptides revealed substantially reduced fluctuations compared to the whole complexes (Figure 1B and 1C). Although transient increases in protein RMSD were observed (up to 0.35 nm), all systems reached stable conformations after approximately 70 ns. The corresponding average RMSD values are summarized in Table 2.

**Table 2:** The average values of RMSD, Rg, and HB of the five studied complexes, with their respective standard deviation. The HB values belong to the last 30 ns of the simulations.

**System**	**RMSD-Total (nm)**	**RMSD-Protein (nm)**	**RMSD-Peptide (nm)**	**Rg (nm)**	**HB**
Control	0.23 +/- 0.06	0.13 +/- 0.02	0.14 +/- 0.05	1.60 +/- 0.01	8 +/- 2
P1	0.11 +/- 0.03	0.18 +/- 0.04	0.03 +/- 0.01	1.60 +/- 0.01	6 +/- 1
P2	0.09 +/- 0.03	0.15 +/- 0.03	0.03 +/- 0.01	1.60 +/- 0.01	6 +/- 1
P13	0.19 +/- 0.05	0.13 +/- 0.02	0.11 +/- 0.04	1.59 +/- 0.01	10+/-1
P23	0.26 +/- 0.09	0.13 +/- 0.02	0.15 +/- 0.05	1.60 +/- 0.01	7 +/- 1

####  All Simulated Complexes Maintained Comparable Structural Compactness.

 Radius of gyration (Rg) analysis showed no significant differences among the five protein–peptide complexes, indicating similar overall folding and compactness throughout the simulations (Table 2).

####  The P13 Peptide Exhibited the Highest Number of Stabilizing Hydrogen Bonds with the Receptor.

 HB analysis performed over the final 30 ns of simulation, when all systems had reached equilibrium, revealed that the P13 peptide formed the highest number of hydrogen bonds (10 interactions), followed by the control (8 interactions) and P23 (7 interactions) peptides (Table 2).

####  Stable HB Distances Confirmed Persistent Protein–Peptide Interactions.

 The distribution of HB distances showed a pronounced peak at 0.25–0.30 nm for all systems, consistent with the presence of the formation of stable hydrogen bonds between the receptor and the designed peptides (Figure 1D).

####  Specific Electrostatic and Hydrophobic Interactions Underpinned the Strong Binding of the P13 Peptide.

 Focusing on the interactions of the P13 peptide, Glu11, Arg12, and Arg14 form hydrogen bonds with Ser153, Ser155, Asp219, Thr221, Glu256, and His258 of the receptor (Figure 2). Furthermore, five residues of the P13 peptide and six receptor residues participated in hydrophobic interactions, further stabilizing the complex.

**Figure 2:**
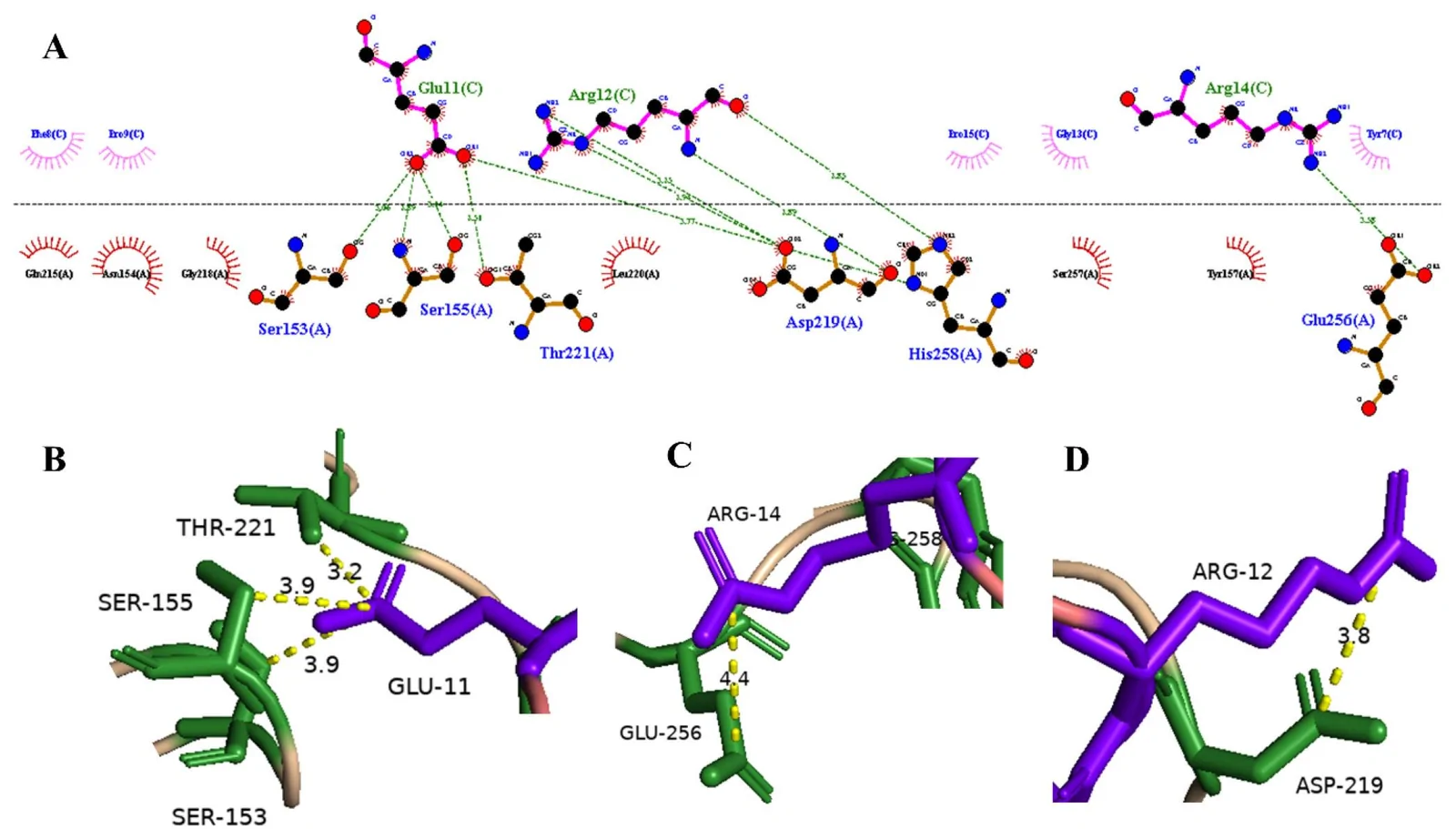
(A) Schematic representation of interactions between integrin α2 and the P13 peptide generated using LigPlot. (B–D) Ribbon representation of the P13 peptide (violet), highlighting interacting residues within the receptor binding site

####  Binding Free Energy Calculations Identified P13 as the Strongest-Binding Peptide

 MM-PBSA analysis revealed that the P13 peptide exhibited the most favorable binding free energy (−240.89 ± 9.14 kJ/mol), compared with the control (−157.68 ± 22.78 kJ/mol) and P23 (−116.71 ± 11.49 kJ/mol) peptides. In all complexes, electrostatic interactions were the dominant contributors to binding energy (Table 3).

**Table 3:** The average values of binding free energy (ΔGGAS) and its components: van der Waals (ΔVDWAALS) and electrostatics (ΔEEL) energies.

**Energy Component**	**Control**	**P1**	**P2**	**P13**	**P23**
**Δ**VDWAALS (kJ/mol)	-35.05 ± 1.04	-27.09 ± 2.84	-26.46 ± 0.28	-31.01 ± 1.02	-30.38 ± 2.19
**Δ**EEL (kJ/mol)	-122.63 ± 22.58	-41.67 ± 32.67	-108.56 ± 18.86	-209.88 ± 8.31	-86.33 ± 10.85
**Δ**GGAS (kJ/mol)	-157.68 ± 22.78	-68.76 ± 32.93	-135.02 ± 19.12	-240.89 ± 9.14	-116.71 ± 11.49

###  Cell Viability 

####  Low Concentrations of the Synthetic Peptide Were Biocompatible with HDF Cells, Whereas Higher Doses Induced Cytotoxicity

 MTT assay results demonstrated that peptide concentrations up to 1.25 μg/mL maintained high HDF viability (≥ 90%) across all evaluated time points (24, 48, and 72 h), showing no significant difference from the control group (Figure 3a). Conversely, higher peptide concentrations (2.5–10 μg/mL) induced a pronounced, time-dependent reduction in viability, with cell viability declining below 50% at 5 and 10 μg/mL, where the highest dose approached complete cytotoxicity.

**Figure 3:**
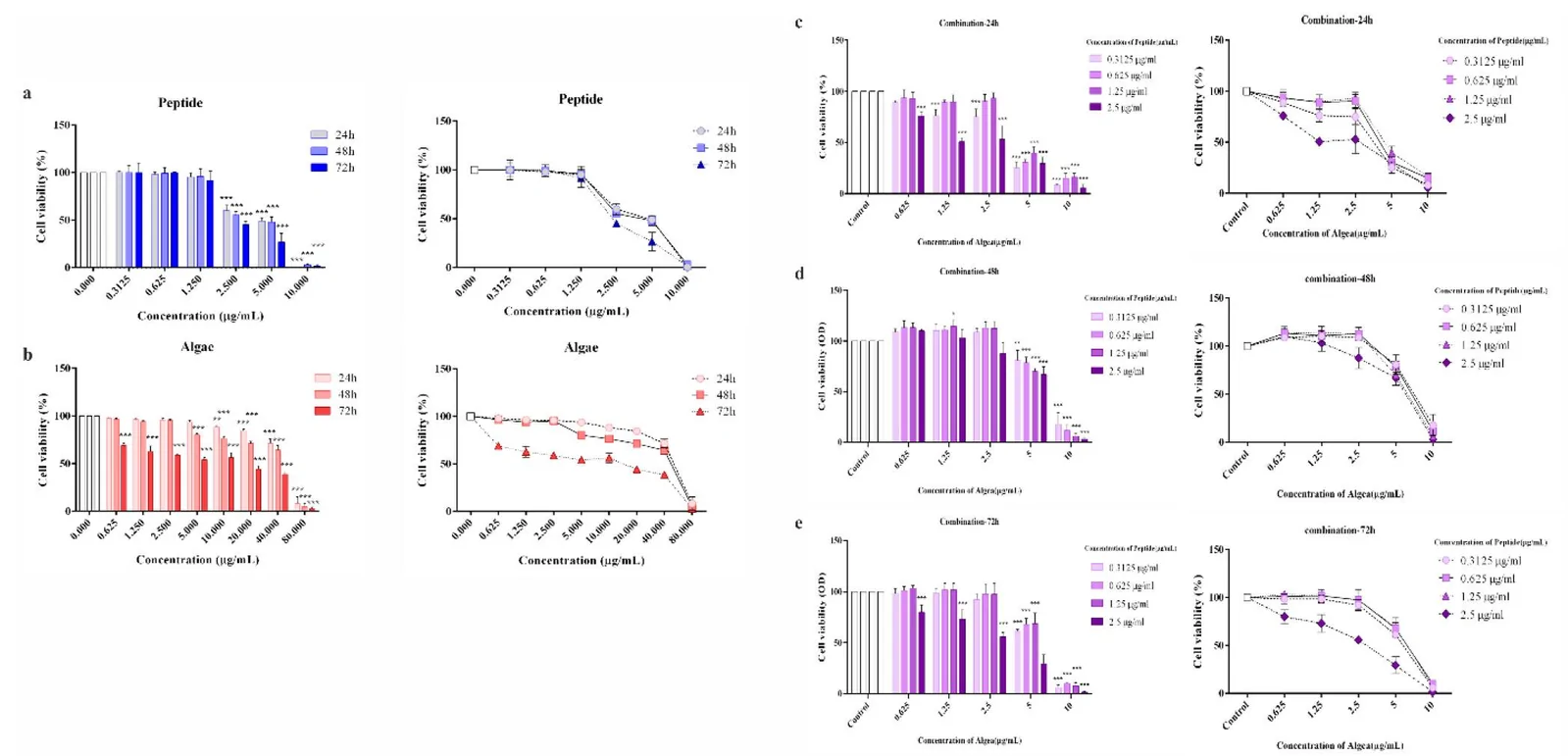
Effects of the synthetic peptide and *C. vulgaris* extract on the viability of human dermal fibroblasts (HDF) assessed by MTT assay. (a) Cells were treated with peptide alone (0–10 µg/mL) for 24, 48, and 72 h. (b) Cells treated with *C. vulgaris* extract alone (0.625–80 µg/mL) for 24, 48, and 72 h. (c-e) The purple bar plots represent combined treatments, with different concentrations of algae extract (0.625–10 µg/mL) on the X-axis and varying peptide concentrations (0.3125–2.5 µg/mL) represented by the column groups, over 24, 48, and 72 h. Cell viability is expressed as a percentage relative to untreated control cells. Data are presented as mean ± SD (n = 3 independent experiments). Statistical comparisons were performed against the untreated control at the corresponding time point. Concentrations selected for subsequent experiments are indicated

####  C. Vulgaris Extract Enhanced Cell Viability at Low Doses But Exhibited Dose- and Time-Dependent Toxicity at Higher Concentrations

 Exposure to low concentrations of algae extract (0.625–5 µg/mL) resulted in near-complete cell viability after 24 h (Figure 3b). However, increasing concentrations and prolonged exposure times significantly reduced HDF viability. Intermediate concentrations (10–40 µg/mL) were well tolerated at 24 h (> 80% viability) but became cytotoxic at later time points, while the highest concentration (80 µg/mL) markedly impaired viability at all time points. Overall, lower doses of algae extract were more effective in supporting cell survival, consistent with previous reports on *C. vulgaris* extracts.^13^

####  Combination Treatments Revealed A Narrow Therapeutic Window with Dose- and Time-Dependent Synergistic Cytotoxicity

 Based on individual dose–response analyses, low-to-moderate concentrations of peptide and algae extract were selected for combination studies. At 24hours, a slight reduction in cell viability was observed in some treatment combinations, likely due to transient cytotoxicity caused by high peptide doses or local aggregation of algal particles. However, at 48 and 72 hours, cell viability significantly increased compared with the control group, indicating a time-dependent recovery and stimulatory effect. Notably, *C. vulgaris* extract alone caused a decline in viability at higher concentrations, while the combined treatment reduced this cytotoxic effect and enhanced proliferation, suggesting an interaction between the two agents and underscoring the importance of careful dose optimization.

###  Gene Expression Analysis

####  Peptide and Algae Extract Treatments Differentially Modulated Collagen Gene Expression in HDF Cells

 qPCR analysis revealed significant regulation of *COL1A1*, *COL1A2*, and *COL3A1 *expression following treatment with peptide, algae extract, and their combinations (Figure 4).

**Figure 4:**
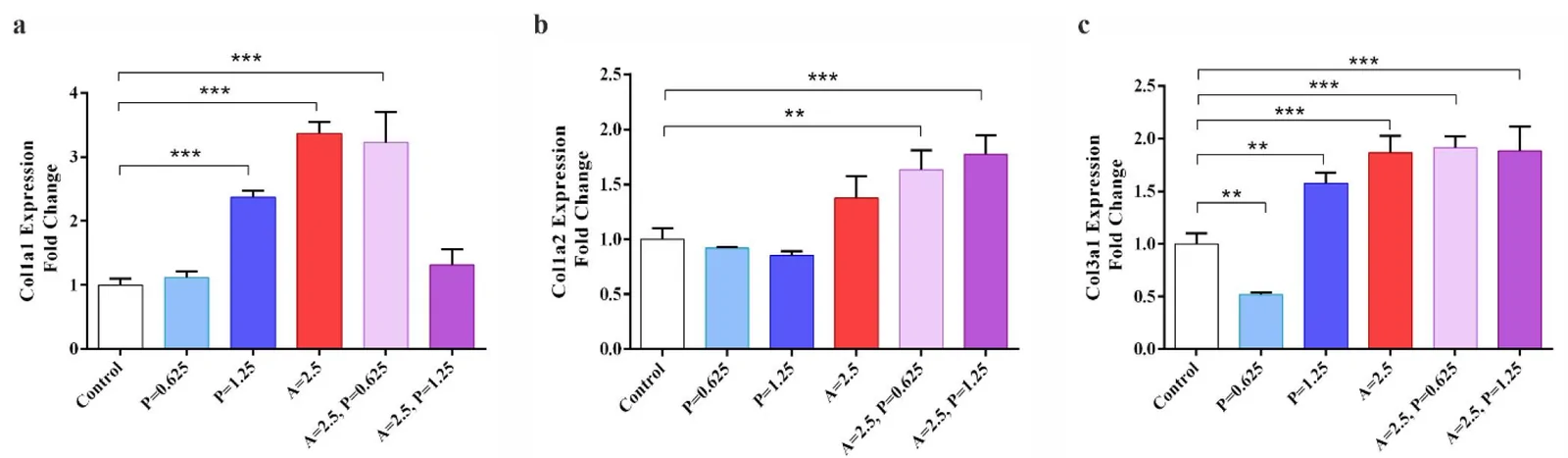
Relative mRNA expression levels of *COL1A1* (a), *COL1A2* (b), and *COL3A1* (c) in human dermal fibroblast (HDF) cells following treatment with peptide (P), algae extract (A), and their combinations (A+P). Cells were treated for 48 h with peptide (0.625 or 1.25 µg/mL), algae extract (2.5 µg/mL), or their corresponding combinations. Gene expression was quantified by RT-qPCR and normalized to the untreated control group using an appropriate internal reference gene. Results are presented as fold change relative to control ± SD. Transcript levels were normalized to the untreated control group and are presented as fold change ± SD. Data represent the mean of two independent experiments; each was performed in triplicate. Statistical significance was determined relative to the control using One-Way ANOVA followed by Tukey’s post hoc test; ∗*P* < 0.05, ∗∗*P* < 0.01, ∗∗∗*P* < 0.001). (*P < 0.05, **P < 0.01, ***P < 0.001)

####  Algae Extract and Low-Dose Peptide Combinations Robustly Upregulated COL1A1 Expression

*COL1A1* expression was significantly increased by peptide at 1.25 µg/mL (~2.5-fold), algae extract alone (~3.5-fold), and the combination of algae with 0.625 µg/mL peptide (~3.2-fold) compared with untreated controls (*P* < 0.001; Figure 4a). In contrast, higher peptide concentrations in combination treatments resulted in attenuated induction, suggesting a dose-dependent inhibitory or saturation effect.

####  Algae Extract Was the Most Potent Inducer of COL1A2 Expression

 The strongest upregulation of *COL1A2* (~2.1-fold) was observed following treatment with algae extract alone (Figure 4b). Combination treatments and peptide at 1.25 µg/mL also significantly increased expression (*P* < 0.01–0.001), whereas peptide at 0.625 µg/mL had minimal impact.

####  Expression of COL3A1 Was Selectively Enhanced by Higher Peptide Doses and Combination Treatments

 While peptide at 0.625 µg/mL suppressed *COL3A1* expression (~0.6-fold), higher peptide concentration, algae extract, and both combination treatments significantly increased expression (~1.8–2-fold, *P* < 0.001; Figure 4c).

 Overall, algae extract exerted a strong pro-collagen effect, with peptide co-treatment modulating gene expression in a dose- and gene-specific manner.

###  Scratch Assay

####  Combined Peptide and Algae Treatment Markedly Enhanced Fibroblast Migration in Vitro

 Fibroblasts are crucial cells that play a key role in the proliferation phase of wound healing.^14^ During the healing of cutaneous wounds, both cell proliferation and migration are critical processes in wound repair. We used a scratch assay to examine the impact of our synthetic peptide and *C. vulgaris *extract on HDF migration (Figure 5a). After 12 h, a noticeable reduction in the scratch area was observed, indicating that cell migration had begun. The algae + peptide group displayed a greater decrease in wound area compared to the other groups.

**Figure 5:**
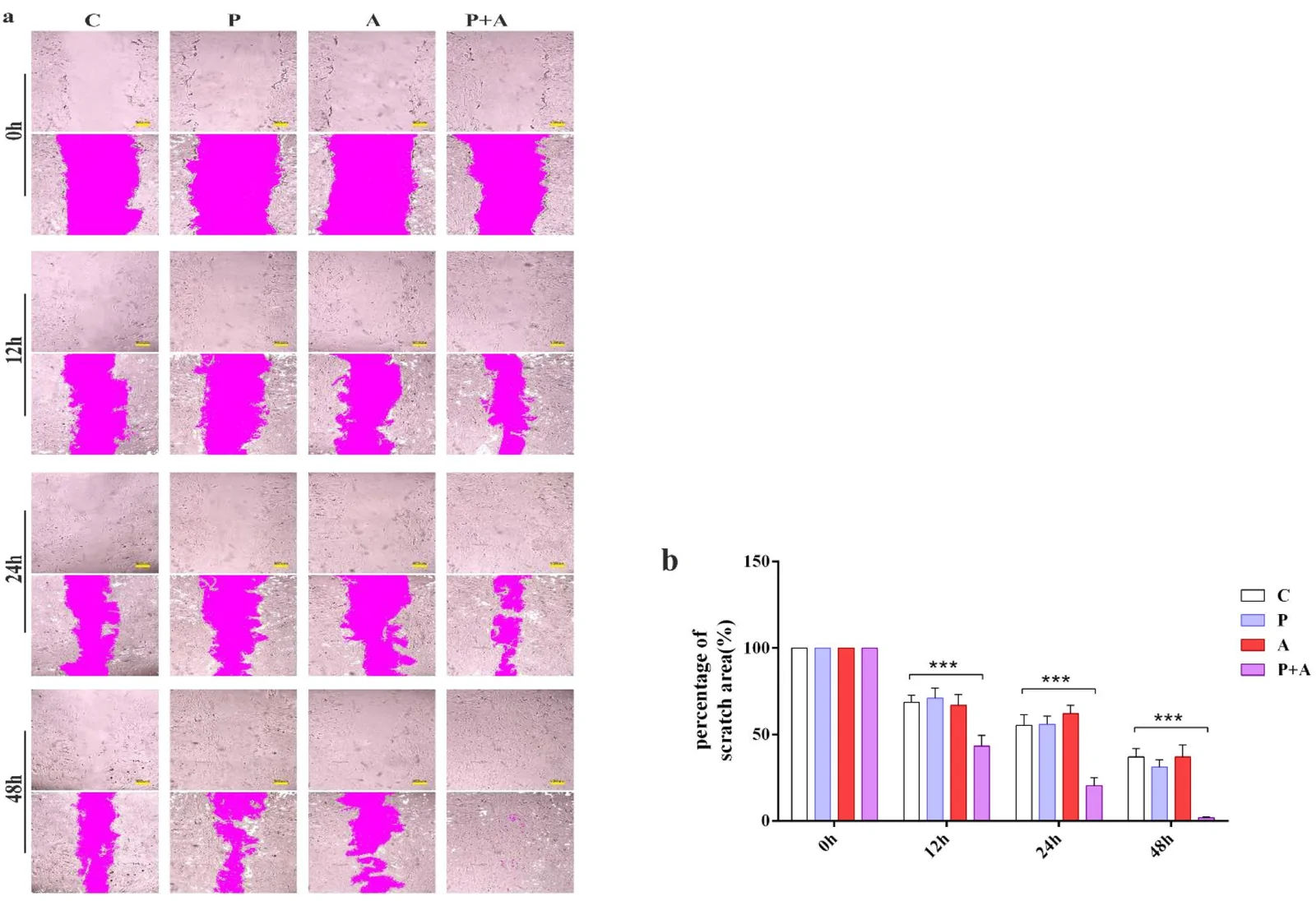
(A) Quantitative analysis of scratch wound closure in human dermal fibroblasts (HDF) at 0, 12, 24, and 48 h following treatment with control, peptide, algae, and algae + peptide (scale bar = 400 µm). (B) Statistical comparisons were performed between treatment groups at each time point and relative to baseline (0 h)

####  The Algae–Peptide Combination Accelerated Wound Closure More Effectively Than Individual Treatments

 At 24 h, cell migration was substantially increased in the combination group compared with peptide-, algae-, or control-treated cells. By 48 h, the algae-peptide-treated monolayers displayed near-complete wound closure, whereas the control group retained a pronounced gap (Figure 5a).

####  Quantitative Analysis Confirmed the Superior Pro-Migratory Effect of the Combination Treatment

 Quantitative analysis of the remaining wound area demonstrated that the combined treatment significantly enhanced fibroblast migration compared with individual treatments and control (Figure 5b). This supported the hypothesis that these compounds accelerated wound healing by activating cellular repair mechanisms.

###  In Vivo Study of Wound Healing Potential

####  Topical Application of the Peptide–Algae Combination Significantly Accelerated Wound Closure in Vivo

 Based on the enhanced migration observed *in vitro*, we further evaluated the wound healing potential of the combination *in vivo*. In a full-thickness excisional wound model, all groups exhibited comparable wound areas at day 0 (*P* > 0.05; Figure 6). From day 3 onward, wounds treated with the peptide–algae combination showed a significantly greater reduction in wound area compared with individual treatments and the scrambled control (*P* < 0.01). This enhanced healing response persisted throughout the observation period.

**Figure 6:**
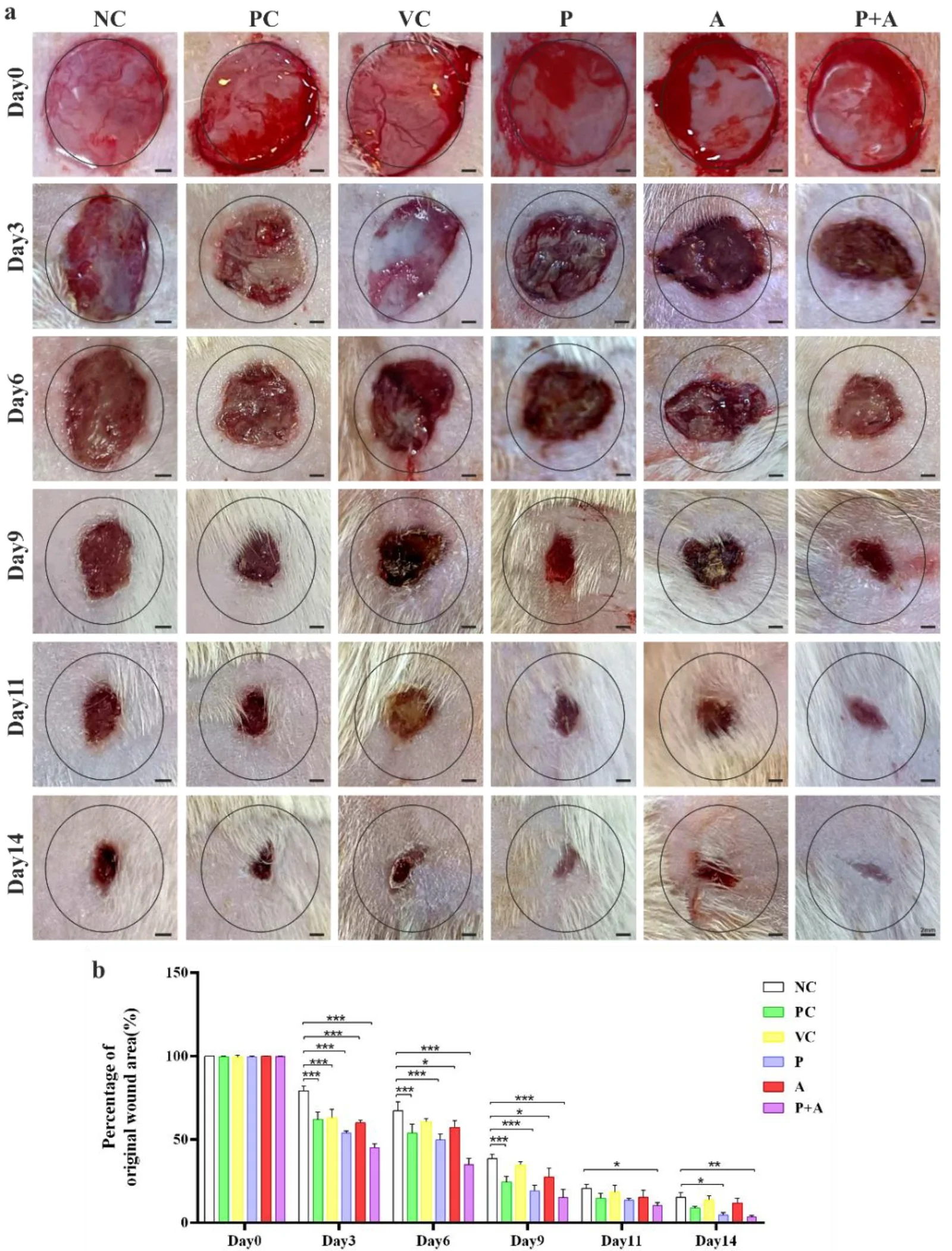
*In vivo* evaluation of wound healing following treatment with synthetic peptide, algae extract, and their combination. (a) Representative photographs of wound contraction in rats treated with Control, commercial wound dressing (positive control), Vaseline (Vehicle control), peptide, algae extract, and peptide + algae on postoperative days 3, 6, 9, 11, and 14 (scale bar = 2 mm; identical magnification). (b) Quantitative analysis of wound size ratio in each group on days 0, 3, 6, 9, 11, and 14. Data are presented as means ± SD

####  The Peptide–Algae Combination Outperformed Individual Treatments Across the Healing Timeline

 At days 3 and 6, the combination treatment induced marked wound contraction, exceeding the effects of peptide or algae alone and closely matching the efficacy of the commercial wound dressing. On day 9, the peptide and algae combination and commercial wound dressing groups showed a significant decrease in wound area compared with other groups (*P* < 0.05). The peptide and algae groups also showed improvement, but to a lesser extent. The scrambled control group exhibited delayed wound closure compared with the treated groups. On day 11, the peptide and algae combination treatment increased wound closure. The commercial wound dressing remained effective, though slightly behind. The individual treatments (peptide and algae) continued to improve the healing, while the scrambled control remained the least effective. On day 14, the peptide and algae combination showed the highest wound closure, with only a minimal wound area remaining, followed by hair growth a few days after the wound closed. Hair growth in the wound area shows the regeneration process, and the skin condition has begun to return to normal.^15^ The commercial wound dressing also showed near-complete healing. The peptide and algae treatments significantly accelerated the wound healing process, but did not match the efficacy of the combination treatment. The scrambled control continued to show the slowest healing rate.

 Overall, the peptide–algae combination produced the most rapid and complete wound healing, while individual treatments accelerated repair to a lesser extent (Figure 6a, b).

###  Histomorphometry Study

####  The Peptide–Algae Combination Markedly Enhanced Collagen Deposition During Wound Remodeling

 Masson’s Trichrome staining revealed progressive increases in collagen density over time in treated wounds (Figure 7). No significant differences were observed among groups at day 3 (*P* > 0.05). By day 6, collagen deposition increased in all treated groups except the negative and scrambled controls.

**Figure 7:**
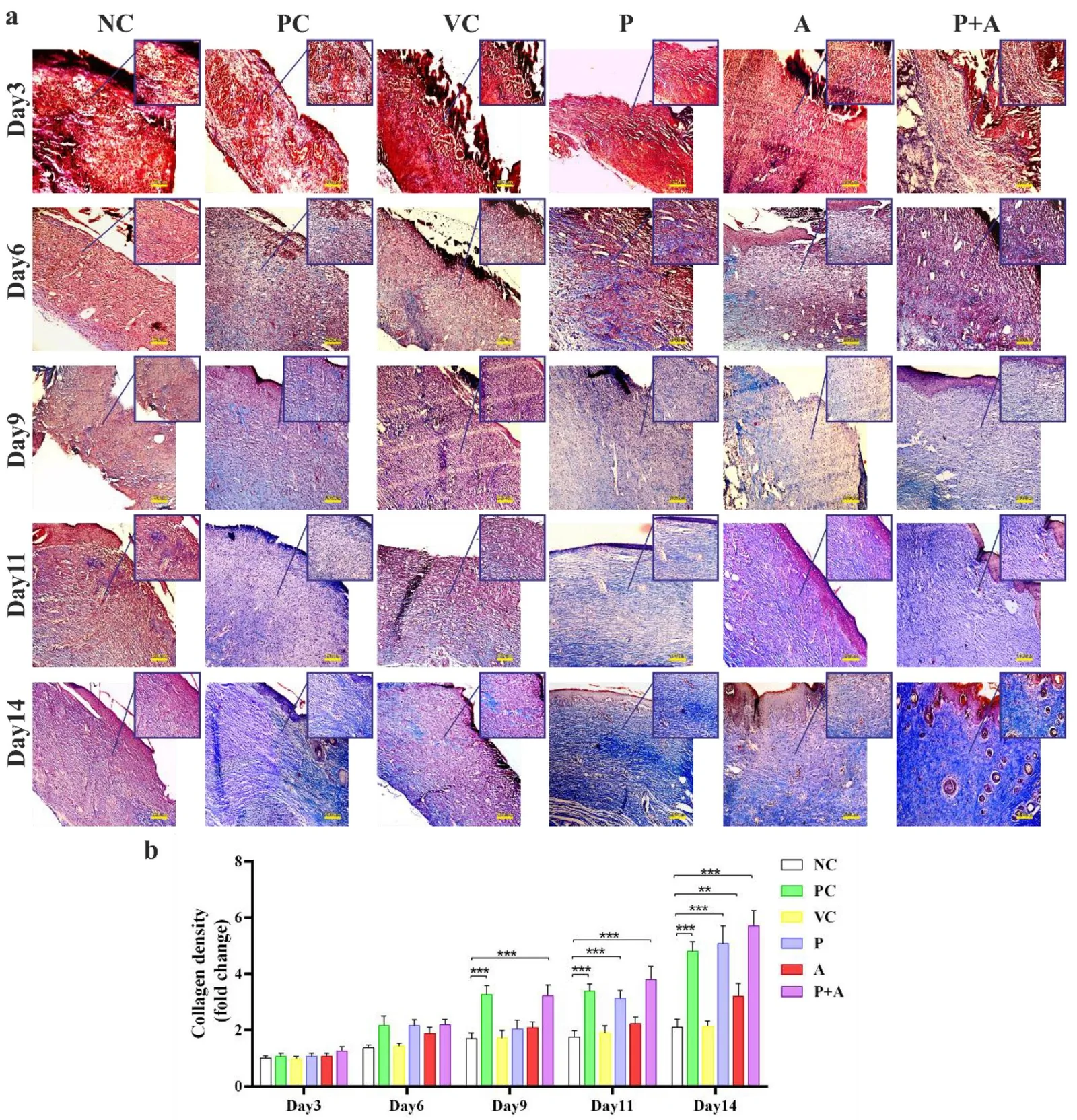
Histological evaluation of excisional wound healing following treatment with peptide, algae, peptide + algae, and commercial wound dressing compared with untreated control rats. (a) Masson’s trichrome staining of full-thickness wound sections collected on days 0, 3, 6, 9, 11, and 14 from control, peptide, algae extract, peptide + algae, and commercial dressing-treated groups (scale bar = 20 µm). (b) Quantitative analysis of collagen deposition measured using ImageJ software. Data are presented as mean ± SD (n = 3 per group)

 Quantitative histological analysis demonstrated a progressive increase in collagen density in the combined *C. vulgaris* extract + peptide group compared with the negative control at all evaluated time points. Collagen content rose from 33.44 ± 3.30 % at day 3 to 151.05 ± 11.90 % at day 14, whereas the control exhibited slower deposition (26.42 ± 1.9 % at day 3 and 55.56 ± 6.33 % at day 14). The differences were statistically significant at all time points (*P* < 0.01; detailed values in Table 4), confirming enhanced collagen formation induced by the combined treatment.

**Table 4:** Quantitative histological analysis in the combined *C. vulgaris* extract + peptide group compared with the negative control at all evaluated time points.

**Group**	**Time Point**	**Collagen Density (Mean±SD, % Area)**	***P*** **-value (vs. Control at Same Time Point)**
Alg. + Pep.	3	33.44 ± 3.30	0.0004
	6	57.92 ± 4.21	0.004
	9	85.31 ± 8.23	0.003
	11	100.62 ± 10.35	0.002
	14	151.05 ± 11.90	0.0006
Neg cont.	3	26.42 ± 1.9	
	6	36.22 ± 2.35	
	9	45.02 ± 4.24	
	11	46.47 ± 4.94	
	14	55.56 ± 6.33	

####  Combination Treatment Induced Superior Collagen Accumulation at Later Healing Stages

 At days 9 and 11, the peptide–algae group exhibited significantly higher collagen density than individual peptide or algae treatments (*P* < 0.01), exceedingly even the commercial wound dressing at day 11. By day 14, the combination treatment maintained the highest collagen content within the wound bed, followed by the peptide and commercial dressing groups. The algae-alone group showed moderate collagen enhancement, while negative and scrambled controls displayed minimal collagen deposition.

 These findings demonstrate that the peptide–algae combination consistently promotes robust collagen synthesis and deposition, particularly during the maturation phase of wound healing, showing comparable or higher collagen density than the commercial wound dressing at selected time points. Peptide and algae individually promote collagen formation but are less effective than their combination.

###  Protein Expression Analysis 

####  Combined Peptide and Algae Treatment Enhanced Collagen I Synthesis and Maturation in Vivo

 Western blot analysis demonstrated increased expression of collagen type I in response to peptide, algae extract, and their combination at days 3, 7, and 11 (Figure 8). Two distinct bands were detected: a ~140 kDa pro-collagen COL I precursor and a ~90 kDa mature COL I protein (Figure 8A).

**Figure 8:**
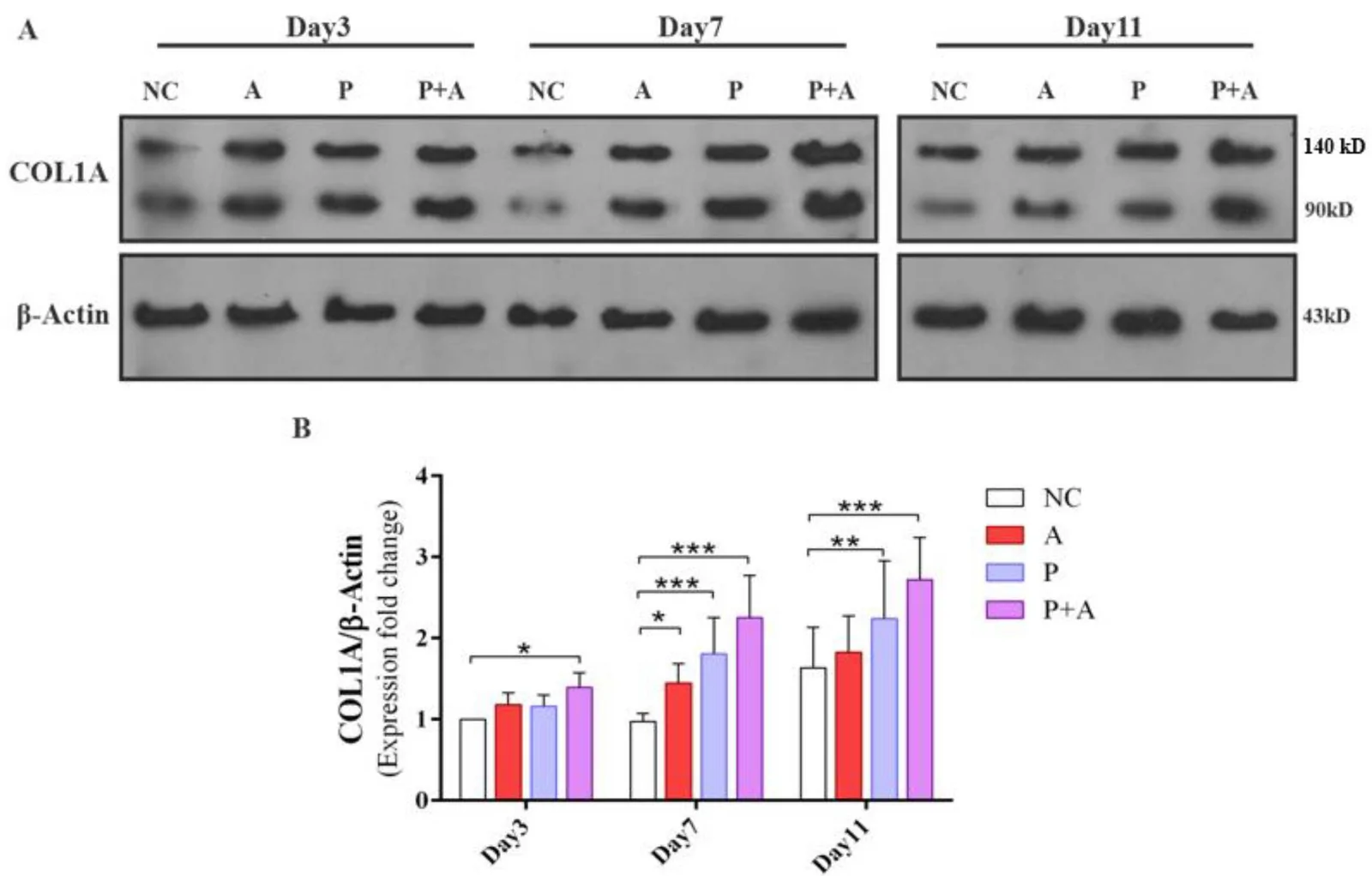
Western blot analysis of COL I protein expression in wound tissues collected on days 3, 7, and 11. (A) Representative immunoblots showing COL I protein levels. Two bands corresponding to ~140 kDa (pro-collagen) and ~90 kDa (mature collagen) were detected in negative control (NC), peptide (P), algae extract (A), and peptide + algae (P+A) groups. β-actin was used as the loading control. (B) Quantitative densitometric analysis of relative COL I protein expression normalized to β-actin. Data are presented as mean ± SD from three independent experiments performed in duplicate

####  The peptide–Algae Combination Induced the Strongest and Most Sustained COL I Expression

 At day 3, the combination treatment markedly increased both pro-collagen and mature COL I levels compared with the control, with a pronounced enhancement of the mature form. By day 7, COL I expression further increased in all treated groups, with the peptide–algae combination nearly doubling the intensity of the mature 90 kDa band relative to control. At day 11, both collagen forms reached their highest levels in the combination group, suggesting enhanced collagen I production and maturation. The peptide-alone group showed a similar but weaker trend, whereas controls consistently exhibited the lowest expression.

 Collectively, these results indicate that the peptide–algae combination promotes both collagen biosynthesis and maturation, thereby supporting effective ECM remodeling during wound healing.

## Discussion

 Wound healing is a tightly regulated biological process that restores skin integrity following injury.^1^ Consequently, the development of therapeutic strategies that accelerate or enhance tissue regeneration remains a central goal in regenerative medicine.^10^ Among recent approaches, protein-based targets with known roles in tissue repair pathways are of particular interest due to their ability to orchestrate key cellular events in the healing cascade.^2^

 Among the various molecular targets, collagen type I plays a pivotal role in the formation and remodeling of the ECM.^3^ In this context, peptides and natural extracts capable of influencing collagen synthesis and deposition present promising avenues for enhancing wound repair.^11,14^

 Integrins, particularly α2β1, are critical mediators of cell–collagen interactions and regulate fibroblast adhesion, migration, and collagen synthesis.^6^ Using MD simulations, we identified peptide P13 as the most favorable ligand for integrin α2, exhibiting superior stability, hydrogen bonding, and binding free energy compared with other designed peptides. The enhanced electrostatic contribution observed for P13 was attributed to its charged amino acid composition. Substituting Tyr7 with a nonpolar amino acid (Gly) in P1 and P2 peptides notably reduced electrostatic energy contribution and total free energy. Additionally, Arg’s greater positive charge compared to Lys enhances electrostatic interactions,^16^ so replacing Arg14 with Lys decreases electrostatic energy and affects the final free-binding energy. Unlike triple-helical collagen mimetics previously reported,^17^ the single-chain peptide design employed here exposes the GFOGER motif, facilitating receptor accessibility and efficient integrin engagement.^18^ Moreover, the short sequence YFPGERGRPG does not require specific orientation to function, unlike full-length proteins, and is less vulnerable to proteolysis or hydrolysis during modifications and after reaching the native environment.^19^ Together, these computational findings supported the selection of P13 as a functional integrin stimulator.

 While our modeling provides a framework for peptide-integrin binding, the exact mechanism of *C. vulgaris* extract in promoting collagen synthesis warrants deeper investigation. The extract is rich in carotenoids and potentially cell-growth-factor-like polysaccharides (CGF), which may directly interact with fibroblast surfaces. Furthermore, the antioxidant capacity of the extract likely mitigates oxidative stress, thereby preserving the viability and synthetic capacity of fibroblasts. We propose that the synergy involves these bioactive components potentiating the integrin signaling cascade (e.g., FAK activation), leading to a significant upregulation of collagen gene expression beyond what the specific peptide interaction alone might achieve.^20-22^

 In vitro experiments demonstrated that both the synthetic peptide and *C. vulgaris* extract were biocompatible at low concentrations, consistent with previous reports.^12,23^ The precise mechanism by which peptides influence cell viability remains unclear; it may be linked to their proliferative effects. Toxicity increased proportionally with algae concentration, possibly due to metals present in the algae. When algae and peptides were combined, toxicity was not observed except at high concentrations, suggesting a possible modulatory interaction, although the underlying mechanism remains unclear. While higher doses induced cytotoxicity in a time- and dose-dependent manner, optimized low-dose combinations preserved cell viability and promoted fibroblast migration. The nonlinear dose–response observed for combination treatments likely reflects receptor saturation or feedback regulation within integrin-mediated signaling pathways. The observed time-dependent changes in the MTT assay suggest that the combination of *C. vulgaris* extract and the peptide exerts a synergistic influence on cell proliferation. The initial decrease in viability at 24 h may reflect an adaptive response to high bioactive compound exposure, followed by activation of intracellular repair and growth pathways over longer durations (48–72 h). This positive shift in viability at later time points indicates that while *C. vulgaris* alone can exert mild cytotoxic effects at higher doses, the presence of the peptide likely modulates this response. Such modulation may occur through integrin-mediated signaling, supporting cell adhesion and survival, alongside antioxidant and CGF-like molecules from the algal extract that counteract oxidative stress. Together, these interactions create a microenvironment favorable for fibroblast activation and collagen synthesis, aligning with the observed enhancement of cell growth in the combined treatment groups.^24,25^

 The current findings highlight a narrow therapeutic window for the synthetic peptide, characterized by high safety margins for HDFs at concentrations up to 1.25 μg/mL (Figure 3a), which contrasts sharply with significant cytotoxicity observed above 5 μg/mL. Critically, the pronounced synergistic cytotoxic effectdemonstrated in combination with the *C. vulgaris* extract (Figures 3c-e) offers a promising avenue for dose optimization and enhanced safetyin a translational setting. Furthermore, comprehensive *in vivo* safety profiling will be paramount to validate the translational potential of this dual-agent approach, ensuring the therapeutic benefit outweighs any narrow safety margins observed *in vitro*.^26,27^

 In the current study, collagen expression was increased *in vitro and in vivo *in cells and animals treated with algae, peptide, and their combination. Based on PCR findings for collagen expression, both individual and combined treatments significantly modulated the transcriptional levels of *COL1A1*, *COL1A2*, and *COL3A1*, with the algae extract showing the most prominent stimulatory effect and supports the idea that bioactive compounds from algae can stimulate ECM production. Peptide treatment, especially at 1.25 µg/mL, also significantly boosted collagen gene expression, indicating that short bioactive peptides may act as signaling molecules to improve fibroblast activity. Nonetheless, the response differed among genes: although *COL1A1* and *COL1A2* were upregulated, the lower peptide concentration (0.625 µg/mL) unexpectedly downregulated *COL3A1*, pointing to a dose-dependent and gene-specific regulatory mechanism.

 The pairing of algae extract with a low dose of peptide (A-2.5 + P-0.625) resulted in a greater increase in *COL1A1* and *COL1A2* expression compared with either treatment alone. This enhanced response may reflect complementary mechanisms. The integrin-targeting peptide likely facilitates receptor engagement and downstream adhesion-related signaling, whereas the algal bioactives may provide metabolic support and ECM–promoting compounds. The simultaneous modulation of cell–matrix interactions and biochemical support could therefore create a more favorable regenerative microenvironment, providing a mechanistic rationale for the combined formulation strategy evaluated in this study.^28,29^ However, adding higher peptide levels (A-2.5 + P-1.25) did not further increase expression and even reduced *COL1A1* induction. This indicated a nonlinear dose-response, possibly due to competitive receptor binding, feedback inhibition, or saturation of intracellular signaling pathways.

 Regarding *COL3A1*, all treatments except the low-dose peptide led to significantly higher expression. The persistent induction by algae extract, whether used alone or combined, further highlighted its potential as a therapeutic agent for promoting dermal collagen remodeling. The decrease caused by peptide alone at 0.625 µg/mL might suggest different regulatory mechanisms for type III collagen, potentially affecting tissue repair and scar formation.

 Peptides from collagen influence cell migration, increasing wound contraction as seen in the study’s treatment groups. To further evaluate the functional impact of peptide, algae extract, and their combination, a scratch assay was conducted to assess cell migration and wound closure over time. As shown in Figure 5a, untreated control cells (C) exhibited limited closure of the wound area even after 48 hours. In contrast, treatment with peptide (P) or algae extract (A) markedly enhanced wound closure, as evidenced by a progressive reduction in the scratch gap at 24 and 48 hours. Notably, the combined treatment (P+A) induced the most substantial wound healing response, with nearly complete closure by 48 hours, indicating an enhanced combined effect on fibroblast migration. This enhanced migration could be attributed to the upregulation of collagen genes observed in our qPCR data (Figure 4), as well as to the possible stimulation of integrin-mediated adhesion and cytoskeletal remodeling pathways. The results are consistent with other studies on the effects of *C. vulgaris*.^30^ These results suggested that the tested compounds not only upregulate ECM components but also enhanced functional repair mechanisms through increased fibroblast motility and proliferation.

 The quantitative increase in collagen density observed from day 3 to day 14 in the combination group suggests a time-dependent stimulation of extracellular-matrix remodeling. The collagen content nearly tripled compared with the control at later stages, highlighting the strong regenerative potential of the C. vulgaris + peptide treatment.

 The presence of two distinct bands at approximately 140 kDa and 90 kDa in the Western blot analysis reflects the detection of both the pro-collagen precursor (pro-COL I) and the mature collagen type I protein (COL I), respectively. The upregulation of the 140 kDa band suggests enhanced synthesis of pro-collagen, while the increase in the 90 kDa band reflects elevated levels of mature collagen type I. Notably, the combined treatment of *C. vulgaris *extract and synthetic peptide led to the most pronounced elevation in both forms, suggesting enhanced collagen production accompanied by increased accumulation of the mature form. This observation is of particular relevance in wound healing, where timely and sufficient collagen deposition is critical for ECM remodeling and tissue regeneration. The strong signal of mature COL I in the combination group, especially on day 11, might indicate a cooperative effect of the peptide and algal bioactives on collagen biosynthesis. These findings align with previous reports highlighting the roles of bioactive peptides and microalgal extracts in enhancing skin repair through modulation of fibroblast activity and ECM production.^31-33^

 Monitoring changes in the wound area can accurately mark healing and contraction rates. The concentrations used for topical administration in the *in vivo* model were determined through preliminary dose optimization, guided by the effective and non-cytotoxic ranges identified *in vitro*. Notably, the nominal concentrations applied *in vivo* were higher than those used in cell culture. This difference reflects the distinct pharmacokinetic and microenvironmental conditions of an open wound compared with a controlled *in vitro* system. In the wound bed, bioactive compounds are subject to dilution by exudate, restricted diffusion within the semi-occlusive Vaseline base, proteolytic degradation by wound-associated enzymes, and partial physical loss during dressing changes. Consequently, the effective local concentration at the cellular interface is substantially lower than the applied concentration. Lower doses tested in preliminary experiments failed to outperform the vehicle control, indicating the presence of a biological threshold concentration required to activate integrin-mediated signaling and downstream collagen synthesis *in vivo*. The selected concentrations (0.625 mg/mL for peptide and 0.4 mg/mL for algal extract) were therefore chosen to ensure sufficient local bioavailability while remaining within a range that did not produce observable irritation, delayed healing, or systemic adverse effects.

 Rats treated with algae + peptide showed maximum healing, comparable to commercial products. While peptide and algae alone expedited healing, their combined effects were more significant. Peptide treatment may promote fibroblast activity and collagen synthesis, thereby contributing to accelerated healing. Findings align with studies on GFOGER peptides and algae.^11,34-35^ *C. vulgaris* proteins aid healing by forming granulation tissue and stimulating fibroblasts, increasing collagen for quick recovery.^15^ Syarina et al. reported microalgae’s efficacy due to phytochemicals beneficial for chronic wounds.^36^ However, *C. vulgaris*’s impact on collagen synthesis in human fibroblasts was unclear. Using *C. vulgaris* extracts in our study enhanced compounds that support tissue repair and remodeling.

 A rapid collagen increase indicates faster tissue regeneration through fibroblast activation and collagen deposition, which consequently has a significant influence on the remodeling phase.^37^ Collagen staining and western blotting showed that algae + peptide treatment promoted collagen synthesis greater than the individual effect, leading to accelerated granulation tissue formation and wound closure without chronic infection or prolonged healing, often seen with overstimulation in early healing stages.^38^ These findings align with other studies on the effects of peptide and algae on collagen.^39-41^

## Conclusion

 Impaired wound healing continues to be a major health problem that predisposes to infections, long-term morbidity, and mortality, particularly in high-risk patients who may consequently suffer from unhealing skin ulcers or even amputation.^14^ A product with a healing purpose must contain compounds that favor or accelerate the natural healing process. This study demonstrated that a collagen-motif-containing synthetic peptide, particularly when combined with *C. vulgaris *extract, significantly enhanced wound healing by promoting fibroblast migration, upregulating collagen gene expression, and increasing collagen deposition *in vivo*.

 Importantly, the peptide–algae combination outperformed individual treatments and showed comparable and, at selected time points, higher efficacy than a commercial wound dressing, highlighting its therapeutic potential. While the findings provided preclinical evidence supporting safety and efficacy, this study was limited to an acute rat wound model. Therefore, further investigations in chronic and disease-associated wound models, along with mechanistic validation and long-term safety assessments, are required before clinical translation. The rat wound healing experiment in this study was limited to 10 animals. While acceptable for preliminary work, the small sample size reduces statistical power. The future studies will be focused on larger cohorts. Besides, the other limitation of this study is the lack of epithelial thickness data, which could provide additional insight into the analyzed outcomes

 Overall, this work introduced a novel bioinspired peptide–microalgae platform as a promising and rational strategy for advanced wound-healing applications.

## Competing Interests

 The authors declare no conflict of interest.

## Data Availability Statement

 The authors will make the raw data supporting this article’s conclusion available upon request.

## Ethical Approval

 All experimental procedures were approved by the Research Ethics Committee of Tarbiat Modares University (Approval No.: IR.MODARES.REC.1400.281).
